# Evaluation of the Effect of Left Nostril Breathing on Cardiorespiratory Parameters and Reaction Time in Young Healthy Individuals

**DOI:** 10.7759/cureus.22351

**Published:** 2022-02-18

**Authors:** Sheela Bargal, Vivek Nalgirkar, Anant Patil, Deepak Langade

**Affiliations:** 1 Physiology, DY Patil deemed to be University School of Medicine, Navi Mumbai, IND; 2 Pharmacology, DY Patil deemed to be University School of Medicine, Navi Mumbai, IND

**Keywords:** parasympathetic tone, sympathetic response, high blood pressure, respiratory rate, yoga research

## Abstract

Background: Pranayama, a yogic breathing practice, produces several physiological responses in healthy individuals. Hypertension is a critical and booming public health challenge all over the world. Yoga is one of the effective methods to reduce blood pressure (BP) and pulse rate (PR).

Objective: To evaluate the effect of left nostril breathing (LNB) exercise on cardiorespiratory parameters and reaction time (RT) in young healthy individuals.

Materials and methods: In this study, 106 young healthy individuals between 18 and 25 years of age were included. The systolic blood pressure (SBP), diastolic blood pressure (DBP), pulse rate (PR), respiratory rate (RR), vital capacity (VC), peak expiratory flow rate (PEFR), and reaction time (RT) of volunteers were recorded at baseline (before exercise) and after two weeks of left nostril breathing exercise performed 45 minutes daily.

Results: There was a significant reduction in SBP (126.64 ± 15.51 mmHg versus 116.29 ± 11.91 mmHg; Cohen’s d (effect size): 0.87; p < 0.0001)), DBP (76.57 ± 14.87 mmHg versus 71.07 ± 11.39 mmHg; Cohen’s d: 0.48; p < 0.0001), PR (85.32 ± 15.44/minute versus 81.77 ± 13.02/minute; Cohen’s d: 0.27; p < 0.0001), and RR (14.26 ± 2.15/minute to 13.17 ± 2.03/minute; Cohen’s d: 0.54; p < 0.0001). A significant increase was observed in VC (3.42 ± 0.62 versus 3.67 ± 0.65; Cohen’s d: 0.39; p < 0.0001) and PEFR (467.81 ± 46.66 versus 498.29 ± 51.50; Cohen’s d: 0.59; p < 0.0001). There was a significant decrement in auditory reaction time (ART) (164.36 ± 27.20 ms versus 143.84 ± 20.32 ms; Cohen’s d: 0.85; p < 0.0001) and visual reaction time (VRT) (190.25 ± 31.48 ms versus 163.75 ± 21.72 ms; Cohen’s d: 0.98; p < 0.0001). There was no significant change in maximum heart rate (MHR) after cardiorespiratory activity (CRA) (p > 0.434).

Conclusion: Left nostril breathing is associated with a decrease in cardiovascular parameters and an increase in VC and PEFR. This technique may be useful for putting up a fight against the stress and strain of daily life. This simple exercise may also be a beneficial adjuvant to pharmacological therapy in hypertensive patients.

## Introduction

With the emerging lifestyle modifications, there has been an increase in the risk of mental stress, hypertension, and other cardiovascular diseases even in younger individuals. The increased incidence of these illnesses could be due to the imbalance between sympathetic and parasympathetic stimulation [1]. Control or modification of breathing or breathing exercises such as pranayama is suggested to have beneficial effects for controlling such diseases. In addition to the specific effects on respiratory functions, several psychophysical effects of pranayama on the body are known [2].

Pranayama is considered a necessary part of yoga. In the yogic breathing system or pranayama, various types of breathing exercises such as Savitri pranayama, Bhastrika pranayama, Nadi Suddhi pranayama (alternate nostril breathing (ANB)/Anulom-Vilom pranayama), Kapalbhati pranayama, Surya Anuloma Viloma pranayama (right uninostril breathing), Chandra Anuloma Viloma pranayama (left uninostril breathing), Ujjayi pranayama (psychic breath) are popular, which produce several physiological responses in healthy individuals. It has already been reported in the earlier studies that all these types of pranayama or breathing exercises reduced the stress and anxiety score and also influence autonomic, cardiac, and respiratory functions [3-5].

Currently, hypertension, a “psychological classical silent killer,” is the indication of several heart disorders. High blood pressure (BP) would turn out to be a leading worldwide load in the upcoming 15-20 years [6]. Yoga is one of the old and popular adjuvant therapies practiced by many people for the management of high blood pressure [7]. Sympathetic overactivity has been commonly reported to be one of the causes of hypertension [8]. Left nostril breathing (LNB) exercise is one type of pranayama. When inspiration and expiration (one breathing cycle) is accomplished by the left nostril only, with the right nostril closed for that time, this type of breathing practice is called “Chandra Anuloma Viloma” pranayama; this breathing practice will transfer heat to cooling effect [6].

Right nostril breathing (RNB) and LNB are an integral part of pranayama. In pranayama, doing breathing exercises through the left nostril increases parasympathetic activity as left nostril dominance corresponds to “ida” svara. A significant decrease in systolic blood pressure (SBP), diastolic blood pressure (DBP), and mean pulse rate/minute (MPR/minute) has been reported after five minutes of LNB exercise. LNB reduces sympathetic activity [6].

In the nasal cycle of 2-8 hours, there is a predominance of one nostril in breathing over the other. The autonomic nervous system (ANS) has an effect on the nasal mucosa and the nasal cycle in which the hypothalamus is the center [9,10].

The strength of respiratory muscles is improved by pranayama, and it also cleanses respiratory secretions. There is a systematic use of diaphragmatic and abdominal muscles during pranayama, thereby emptying and filling the respiratory apparatus with reduced viscous resistance and elasticity of the lung present during inspiration. Pranayama breathing improves the capability of respiratory muscles and lung compliance. Pranayama acts as a stimulus for various respiratory activities or lung functions as lung surfactants and prostaglandins are released into alveolar spaces, which increases lung compliance [11-13].

Reaction time (RT) is the period between the presentation of a stimulus and the initiation of a response. It is a very susceptible and indirect index of central neuronal processing, and it determines the relationship between sensory and motor systems, their performance, and cortical arousal. RT determines the alertness of a person to quickly detect or respond to slight changes, signals, or influences. There have been reports on the effect of different durations of comprehensive yoga training on RT and that pranayama decreases RT [3,14]. Very few studies can be found determining RTs in medical students.

The present research was therefore undertaken to explore the effect of two-week LNB exercise on cardiorespiratory parameters and reaction time in young healthy volunteers [15].

## Materials and methods

In this interventional study conducted in a medical college, out of 110 participants screened, four were excluded due to age criteria. Finally, a total of 106 volunteers of both genders aged between 18 and 25 years, without any cardiopulmonary disease and not under treatment for any disease, were included. Initial parameters such as the age, height, and weight of the participants were recorded. Body mass index (BMI) was calculated using the height and weight of the participant. The experimental protocol was described to all volunteers, and written informed consent was collected from each student preceding the study. Subjects with past history of any medical disorders including diabetes, arthritis, cardiac disease, auditory or visual defect, smokers, alcoholics, upper respiratory tract infections, or nasal obstruction were excluded from the study. Participants taking any medication or doing regular breathing exercises were also excluded from the study.

The volunteers were instructed not to practice any other exercise or yogic exercises other than the prescribed one during the study period. The participants practiced an LNB session of 45 minutes from 9 am to 9:45 am after light breakfast. The schedule continued for two weeks.

The participants were requested to sit adequately in an easy and steady posture (Sukhasana), keeping the head, neck, and trunk erect, and the eyes should be closed with fingers positioned in chin mudra. The LNB practice should be done in a calm and quiet room. The participants were directed to inhale and exhale through the left nostril only. The LNB exercise included cycles of inspiration and expiration through the left nostril (LN) while the right nostril (RN) is closed by the thumb of the right hand, and breathing should be slowly and maximally through the left nostril only [6,16]. The LNB exercise is practiced (six cycles/minute) for 45 minutes daily for two weeks. The LNB exercise was done under the guidance of a yoga expert teacher. Vital capacity (VC), peak expiratory flow rate (PEFR), systolic blood pressure (SBP), diastolic blood pressure (DBP), pulse rate (PR), auditory reaction time (ART), visual reaction time (VRT), and cardiorespiratory activity (CRA) were recorded at baseline and after two weeks. SBP, DBP, and PR were measured while in a seated position. All the parameters were recorded at the same time of the day to avoid diurnal variations. Vital capacity was measured using a windmill-type spirometer. PEFR was obtained using a mini Wright peak flow meter.

In order to observe the effect on CRA, we used a stationary recliner bicycle. As per the guideline of the American College of Sports Medicine (ACSM), the safe and effective exercise intensities for people with sedentary lifestyles and deconditioned individuals should be low to moderate, i.e., 39%-59% heart rate reserve [17].

Recliner seats were adjusted to match the subject’s height in such a way that a 90° knee flexion occurred while paddling. Warmup was done for five minutes at low intensity. Then, the participants cycled for 10 minutes at low to moderate intensity. The Wrist Blood Pressure Monitor HEM-6181 (Omron Healthcare Manufacturing Vietnam Co. Ltd., Thu Dau Mot, Vietnam) was used to record blood pressure and pulse rate/minute. The target heart rate (THR) at a given intensity was calculated using the Karvonen formula: THR (bpm) = % intensity × ((220-age) - resting heart rate (RHR) + RHR [18,19]. ART and VRT were measured using a reaction time machine [20]. Permission to conduct the study was taken from the Institutional Ethics Committee of Biomedical and Health Research, DY Patil Medical College and Hospital, Navi Mumbai, India (approval number: DYP/IECBH/2019/13).

The sample size was estimated based on the primary study outcome, i.e., the change in SBP after two weeks of breathing exercises. Based on the reported data for change (fall) in SBP with left nostril breathing, a sample size of 110 in each group achieved a power of 80.52% [20]. This sample was adequate to a difference of -2.8 between the null hypothesis that both group means are -1.3 with a known standard deviation of 5.1 and a significance level (alpha) of 0.050 (95% confidence levels) using a two-sided t-test, assuming that the actual distribution is uniform. Continuous data are presented as means and standard deviations (SD). Cohen’s d was calculated for the estimation of effect size. A paired t-test was applied to test the significance. P values of <0.05 were considered statistically significant. SPSS version 23 (IBM Corp., Armonk, NY, USA) was used for statistical analysis.

## Results

The study included a total of 106 healthy volunteers with a mean age of 18.77 years. The other baseline characteristics of the study participants are shown in Table 1.

**Table 1:** Baseline characteristics of the study participants SD: standard deviation

Parameters	Result
Age in years (mean ± SD)	18.77 ± 1.50
Male (n (%))	53 (50%)
Female (n (%))	53 (50%)
Height in centimeters (mean ± SD)	163.70 ± 10.15
Weight in kg (mean ± SD)	60.89 ± 16.35
Body mass index in kg/m^2^ (mean ± SD)	22.64 ± 4.16

There was a significant decrease in SBP, DBP, PR, and RR and a significant increase in VC and PEFR after the LNB exercise. There was no significant change in maximum heart rate (MHR) after two weeks of LNB exercise. A significant decrease in ART and VRT was observed after the LNB exercise (Table 2).

**Table 2:** Effect of LNB exercise on cardiovascular and respiratory parameters in healthy young individuals ART: auditory reaction time; CI: confidence interval; DBP: diastolic blood pressure; LNB: left nostril breathing; MHR: maximum heart rate; PR: pulse rate; RR: respiratory rate; SBP: systolic blood pressure; SD: standard deviation; VRT: visual reaction time

Parameters	Before (mean ± SD)	After (mean ± SD)	Change Δ (mean (95% CI))	t value	P value	Cohen’s d (effect size)
SBP (mmHg)	126.64 ± 15.51	116.29 ± 11.91	10.35 (8.14–12.55)	9.29	0.0001	0.87
DBP (mmHg)	76.57 ± 14.87	71.07 ± 11.39	5.50 (2.92–8.07)	4.24	0.0001	0.48
Pulse rate (beats/minute)	85.32 ± 15.44	81.77 ± 13.02	3.55 (2.00–5.08)	4.57	0.0001	0.27
Respiratory rate (RR/minute)	14.26 ± 2.15	13.17 ± 2.03	1.09 (0.72–1.47)	5.82	0.0001	0.54
VC (L)	3.42 ± 0.62	3.67 ± 0.65	-0.025 (-0.29–-0.21)	-12.32	0.0001	0.39
PEFR (L)	467.81 ± 46.66	498.29 ± 51.50	-30.48 (-34.75–-26.19	-14.12	0.0001	0.59
MHR	147.36 ± 8.43	147.81 ± 5.71	-0.45 (-1.60–-0.69)	-0.78	0.434	0.08
ART (ms)	164.36 ± 27.20	143.84 ± 20.32	20.52 (16.88–24.15)	11.19	0.0001	0.85
VRT (ms)	190.25 ± 31.48	163.75 ± 21.72	26.50 (22.19–30.81)	12.20	0.0001	0.98

## Discussion

The present study was designed to evaluate the effect of the short-term pranayama technique on cardiorespiratory parameters and reaction time in young healthy individuals. A significant increment in PEFR and VC and a significant decrease in ART, VRT, SBP, DBP, RR, and PR with no change in MHR were observed in the study. After breathing exercise through the LN, changes were observed in different cardiovascular and respiratory parameters, which may be a response for balancing sympathetic and parasympathetic activation.

A decrease in RR and an increase in VC and PEFR were consistent with other studies [15,21,22]. During yoga training or any breathing exercise, the lungs inflate and deflate due to regular inhalation and exhalation for an extended period, which causes strengthening and increased endurance of the respiratory muscles [15,21,22].

Yoga training can improve respiratory parameters such as forced expiratory volume (FEV1) and PEFR in students [12]. As reported in another study, pulmonary function tests, such as maximal voluntary ventilation (MVV), forced expiratory volume (FEV1), and forced vital capacity (FVC), improve, and respiratory rate (RR) decreases after practicing pranayama for eight weeks [6]. In our study, PEFR values increased after LNB exercise. It may be due to an increment in thoracic-pulmonary compliance and bronchodilation.

Breathing practice increases the vagal tone of the sinoatrial (SA) and atrioventricular (AV) node and also improves baroreceptor sensitivity, although at a breathing rate of six breaths per minute [23]. The decrease in HR and BP indices following LNB exercise may be due to the above changes that occur because of LNB exercise. It has been reported that the circadian rhythms of HR may be influenced by the coordination between right sympathetic and left parasympathetic dominance [24]. As reported, sympathetic overactivity commonly leads to hypertension [8]. In our study, PR, SBP, and DBP decreased significantly with LNB exercise. These results may be due to the alternative function of the left nostril and right nostril in our body. When the nasal passage is clean and unobstructed by mucus, the right or left nostril is more congested than the other. In the pranayama or yogic system of breathing, the left nostril activates the parasympathetic activity as its dominance corresponds to “ida” svara [25]. Jain et al. have reported that SBP and DBP decrease after 15 minutes of LNB and even after eight weeks of LNB practice [25]. Our findings coincide with the study of Naik et al., who reported that just after five minutes of LNB practice, there is a significant decrease in cardiovascular parameters such as mean pulse rate/minute, SBP, and DBP [6].

In our study, there was no marked difference in MHR after the LNB exercise. When practicing the LNB exercise, there is a significant shortening of the duration of both ART and VRT. After practicing pranayama, the reactivity of RT becomes faster.

In our study, the RT for auditory stimulus and visual stimulus decreases significantly after the LNB exercise, but when the duration of ART and VRT after LNB exercise is compared, the ART duration was shorter than the VRT duration, which means that the ART has a faster RT than the VRT. This result also coincides with the other studies in the literature. The RT for auditory stimulus is fast for any type of given stimulus, as reported in the study of Pain et al. [26]. VRT is relatively 180-200 ms, and ART is around 140-160 ms; this result was detected by Thompson et al. [27]. Visual stimulus takes time to reach the brain, at 20-40 ms, while auditory stimulus takes only 8-10 ms [28].

Our study has limitations in terms of the sample size and the nature of the participants. As our study involved healthy individuals, the results should be carefully extrapolated to the patients. Studies in patients with hypertension or respiratory disorder such as chronic obstructive lung disease may be performed in the future. Additional assessment, such as galvanic skin resistance, would have given a better understanding of the state of sympathetic and parasympathetic functions.

## Conclusions

LNB exercise helps improve cardiorespiratory parameters and reaction time. Therefore, this simple exercise may be useful in modulating the physiological functions of the heart and lungs in healthy individuals. Further studies in hypertensive patients or those with pulmonary disorders with appropriate monitoring may help in understanding the scope of this non-pharmacological management in eligible patients. As per the results of our study, LNB exercise can increase auditory and visual RT, suggesting the potential benefit of this exercise in the healthy younger population. Larger, multicenter studies are necessary to confirm our observations.
